# Immunodetection of Pyruvate Carboxylase Expression in Human Astrocytomas, Glioblastomas, Oligodendrogliomas, and Meningiomas

**DOI:** 10.1007/s11064-023-03856-5

**Published:** 2023-01-20

**Authors:** Eduard Gondáš, Alžbeta Kráľová Trančíková, Katarina Dibdiaková, Tomáš Galanda, Jozef Hatok, Peter Račay, Dušan Dobrota, Radovan Murín

**Affiliations:** 1 Department of Medical Biochemistry, Jessenius Faculty of Medicine in Martin, Comenius University in Bratislava, 036 01 Martin, Slovakia; 2 Biomedical Center Martin, Jessenius Faculty of Medicine in Martin, Comenius University in Bratislava, Martin, Slovakia; 3 Institute of Neuroimmunology, Slovak Academy of Sciences, Bratislava, Slovakia; 4 Department of Neurosurgery, Roosevelt Hospital, Slovak Medical University, Banská Bystrica, Slovakia

**Keywords:** Pyruvate carboxylase, Brain tumor, Glioblastoma, Astrocytoma, Meningioma, Oligodendroglioma

## Abstract

Pyruvate carboxylase (PC) is an enzyme catalyzing the carboxylation of pyruvate to oxaloacetate. The enzymatic generation of oxaloacetate, an intermediate of the Krebs cycle, could provide the cancer cells with the additional anaplerotic capacity and promote their anabolic metabolism. Recent studies revealed that several types of cancer cells express PC. The gained anaplerotic capability of cells mediated by PC correlates with their expedited growth, higher aggressiveness, and increased metastatic potential. By immunohistochemical staining and immunoblotting analysis, we investigated PC expression among samples of different types of human brain tumors. Our results show that PC is expressed by the cells in glioblastoma, astrocytoma, oligodendroglioma, and meningioma tumors. The presence of PC in these tumors suppose that PC could support the anabolic metabolism of their cellular constituents by its anaplerotic capability.

**Supplementary Information:**

The online version contains supplementary material available at 10.1007/s11064-023-03856-5.

## Introduction

The transformed metabolism of cancer cells is adapted to sustain their energy needs and sustain the conversion and/or biosynthesis of the compounds crucial for their growth and division. The cytosolic processes of glucose utilization by glycolysis [1] and pentose-phosphate pathway [2] should be supplemented by functional mitochondrial metabolism, especially reactions of the Krebs cycle [3]. Typical feature of the transformed metabolism of cancer cells is increased production of lactate by intensified flux of glucose through glycolytic pathway, so-called Warburg effect [1, 4, 5]. Even higher flux from glucose derived carbon atoms through pyruvate to lactate is anticipated in the cancer cells their capacity to oxidaze pyruvate is limited due to inhibition of pyruvate dehydrogenase complex. Although, several intermediates of the Krebs cycle are the indispensable substrates for encouraging the biosynthetic reaction. The withdrawal of the Krebs cycle intermediates has to be replenished by an anaplerotic process to maintain mitochondrial metabolism. Only a limited number of reactions is considered to possess the anaplerotic role [6]. Among them, the generation of 2-oxoglutarate from glutamine or glutamate was considered as the most significant anaplerotic process in the cancer cells [7–9]. In this respect, the catabolism of glutamate and glutamine, which are taken up from the cellular milieu, could impart the cells with both carbon skeleton and reduced nitrogen [10]. The recent studies with the cultured cancer cells and on the animal models revealed that also the anaplerotic role of pyruvate carboxylase (PC) could significantly participate in sustaining the metabolism and survival of cancer cells and the growth of tumors [11–17]. PC is mitochondrial protein, which have to be for its molecular activity converted from apoenzym to holoenzyme form by its biotinylation [18].

The carboxylation of pyruvate to oxaloacetate by PC could provide the cells with the capability to convert pyruvate, an intermediate of glycolysis, to a Krebs cycle member [19]. Oxaloacetate could be converted further to several compounds such as phosphoenolpyruvate, citrate, aspartate, malate or pyruvate [20]. Together with aspartate and malate, citrate could impact the cellular metabolism in mitochondrial and cytosolic compartments. Citrate transported into the cytosol is the significant source of the cytosolic acetyl-CoA for synthesizing fatty acids and cholesterol [21], while malate and aspartate also participate in the electron shuttle process [22]. The generation of phosphoenolpyruvate is an essential reaction for sustaining gluconeogenesis in liver and kidney cells [23].

Since anaplerotic reactions profoundly impact cellular metabolism, the possibility of targeting the critical anaplerotic enzymes could provide new therapeutic approaches in managing cancer diseases. While the results of the experiments on the cell cultures and animal models [14, 16, 24, 25] could provide valuable hints about the role of PC as the putative target for prognosis and therapy, only the rigorous testing of the human cancers might confirm the presence of PC in different types of tumors. Therefore, we investigated by the immunoblotting methods the presence of PC among the different types of human brain tumor samples, which were surgically removed from the patients during the therapy. We provide pieces of evidence that PC is expressed in different types of human brain tumors and in the healthy brain.

## Results

The suggested important role of PC in supporting the metabolism and features of cancer cells in culture and animals evokes the question to which extent PC is also expressed among human tumors in vivo. To investigate the presence of PC in several types of brain tumors (Table 1) obtained from the patients after the surgical resections, we applied immunoprobing-based analysis.

**Table 1:** Characteristics of the patients with brain tumors

	Glioblastoma	Astrocytoma	Oligodendroglioma	Meningioma
Number	10	10	7	7
Age (years)	55 ± 17	36 ± 14	33 ± 15	58 ± 11
Gender				
Male/female	3/7	4/6	4/3	3/4

**Fig. 1:**
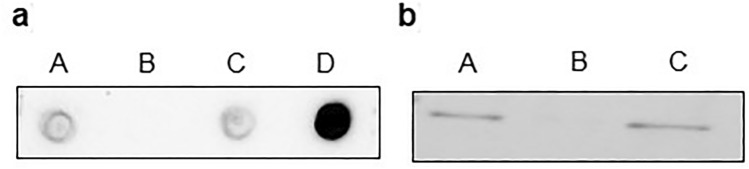
Evaluation of pyruvate carboxylase antibodies in glioblastoma tumor sample. Dot blot (**a**) and western blot (**b**) analysis of PC presence in glioblastoma homogenate (A). The same homogenate was incubated with streptavidin agarose beads (B), and subsequently, the proteins from streptavidin agarose beads were eluted with biotin (C). PC purified from bovine liver (D) was used as a positive control for dot blot. Both immunoprobing assays were performed as described in the Methods part

To exploit the capability of antibodies, those were initially derived against PC isolated from bovine liver [26], also to detect the human ortholog, their specificity was tested. By immunoprobing of the proteins in glioblastoma tumor homogenate, the antibodies gave a positive signal (Fig. 1a/A, b/A), which in the case of Western blot analysis corresponds to the protein with a relative molecular mass of 125 kDa (Fig. S1). The treatment of the homogenate with the streptavidin agarose beads prevented the detection of the signal (Figs. 1a/B, b/B) that reappeared again after elution of proteins from streptavidin agarose beads by applied biotin (Fig. 1b/C, b/C). As a positive control for a dot blot analysis, the purified pyruvate carboxylase from the bovine liver was used (Figs. 1a/D, 2 PC_(pur)_). The biotin eluted fraction was also examined for PC activity. Indeed, the enzymic activity of PC was detected (data not shown).

**Fig. 2:**
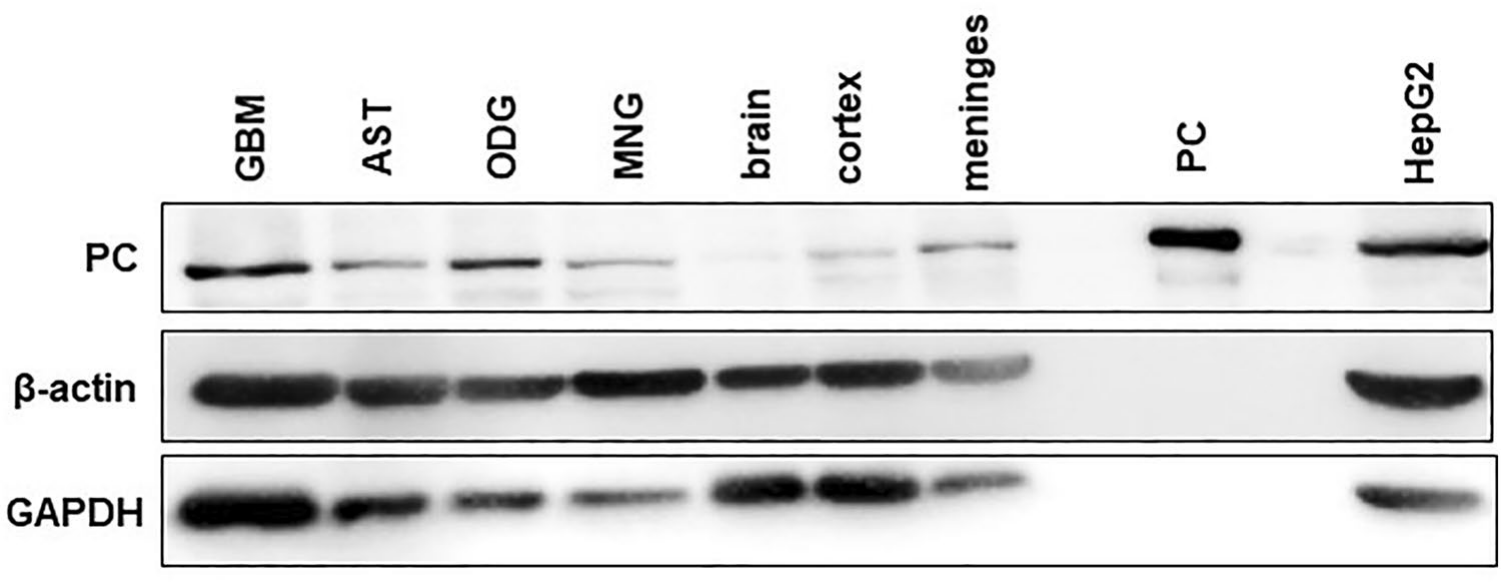
Expression of pyruvate carboxylase in human brain tumors, healthy brain and in gluconeogenetic cells. The western blot analysis of PC expression in glioblastoma (GBM), astrocytoma (AST), oligodendroglioma (OGD), meningioma (MNG), total brain homogenate (brain), cerebral cortex (cortex), cerebral meninges (meninges), purified pyruvate carboxylase (PC_(pur)_), and hepatocarcinoma cell line (HEPG2). The samples were probed with rabbit anti-PC primary antibodies and subsequently with affinity-purified anti-rabbit IgG secondary antibodies covalently linked to horse-radish peroxidase. The generated chemiluminescent signal was recorded (PC). The same membrane was probed with mouse anti-β-actin and mouse anti-GAPDH primary antibodies and subsequently with affinity-purified anti-mouse IgG secondary antibodies covalently linked to horse-radish peroxidase. The generated chemiluminescent signal was recorded (β-actin, GAPDH)

**Fig. 3:**
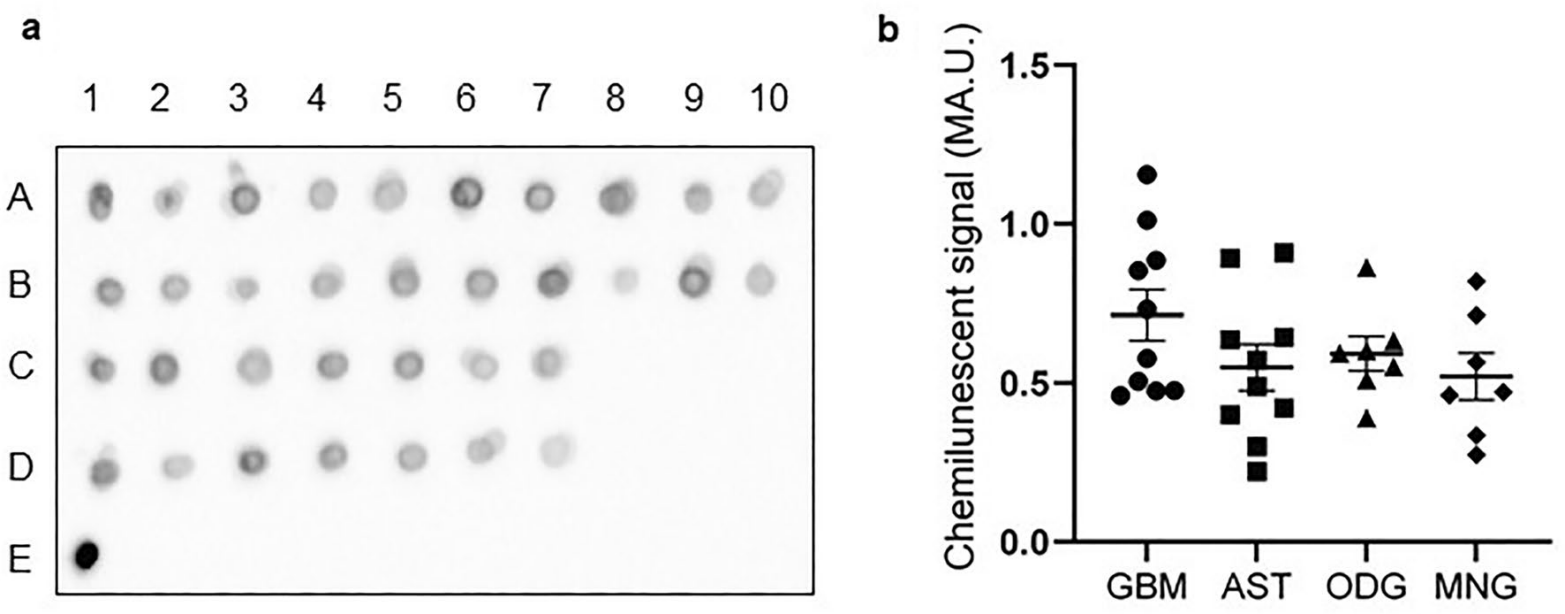
Expression of pyruvate carboxylase among human brain tumors. The presence of pyruvate carboxylase among of lysate proteins derived from glioblastoma (GBM, A1–A10), astrocytoma (AST, B1–B10), oligodendroglioma (ODG, C1–C7), meningioma (MNG, D1–D7), and purified pyruvate carboxylase (E1) samples was detected by dot blot analysis. The obtained chemiluminescent signal was recorded (**a**), quantified, and plotted (**b**)

The Western blot analysis shows that PC is expressed among all of the tested protein extracts obtained from glioblastoma, astrocytoma, oligodendroglioma, and meningioma tumors; and also in protein lysates derived from the total human brain, cerebral cortex, and meninges (Fig. 2). The glioblastoma sample in the blot (Fig. 2; GBM) corresponds to spot A1 in Fig. 3, such as other samples are shown in dot blot: astrocytoma (Fig. 2; AST) in spot B2, oligodendroglioma (Fig. 2; ODG) in spot C4, and meningioma (Fig. 2; MNG) in spot D4 in Fig. 3.Purified bovine pyruvate carboxylase and lysate of hepatocarcinoma cells (Fig. 2) were used as positive controls. Indeed, the estimated relative molecular mass of the visualized bands corresponds mass of PC (125 kDa). Both proteins, GAPDH and β-actin (Fig. 2), were visualized as the loading controls.

The qualitative analysis of the obtained chemiluminescent signal revealed the ubiquitous presence of signal corresponding to PC expression among tested protein extracts prepared from glioblastoma (Fig. 3a, A1–A10), astrocytoma (Fig. 3a, B1–B10), oligodendroglioma (Fig. 3a, C1–C7) and meningioma (Fig. 3a, samples D1–D7) tumors. Purified pyruvate carboxylase was used as a positive control (Fig. 3a, sample E1). The recorded chemiluminescent signal was observable from all samples with varying intensities of quantified signal within the particular groups of the samples (Fig. 3b). Comparison of quantified chemiluminescent signal in different types of tested brain tumors did not revealed any statistical differences in expression levels of PC among them.

**Fig. 4:**
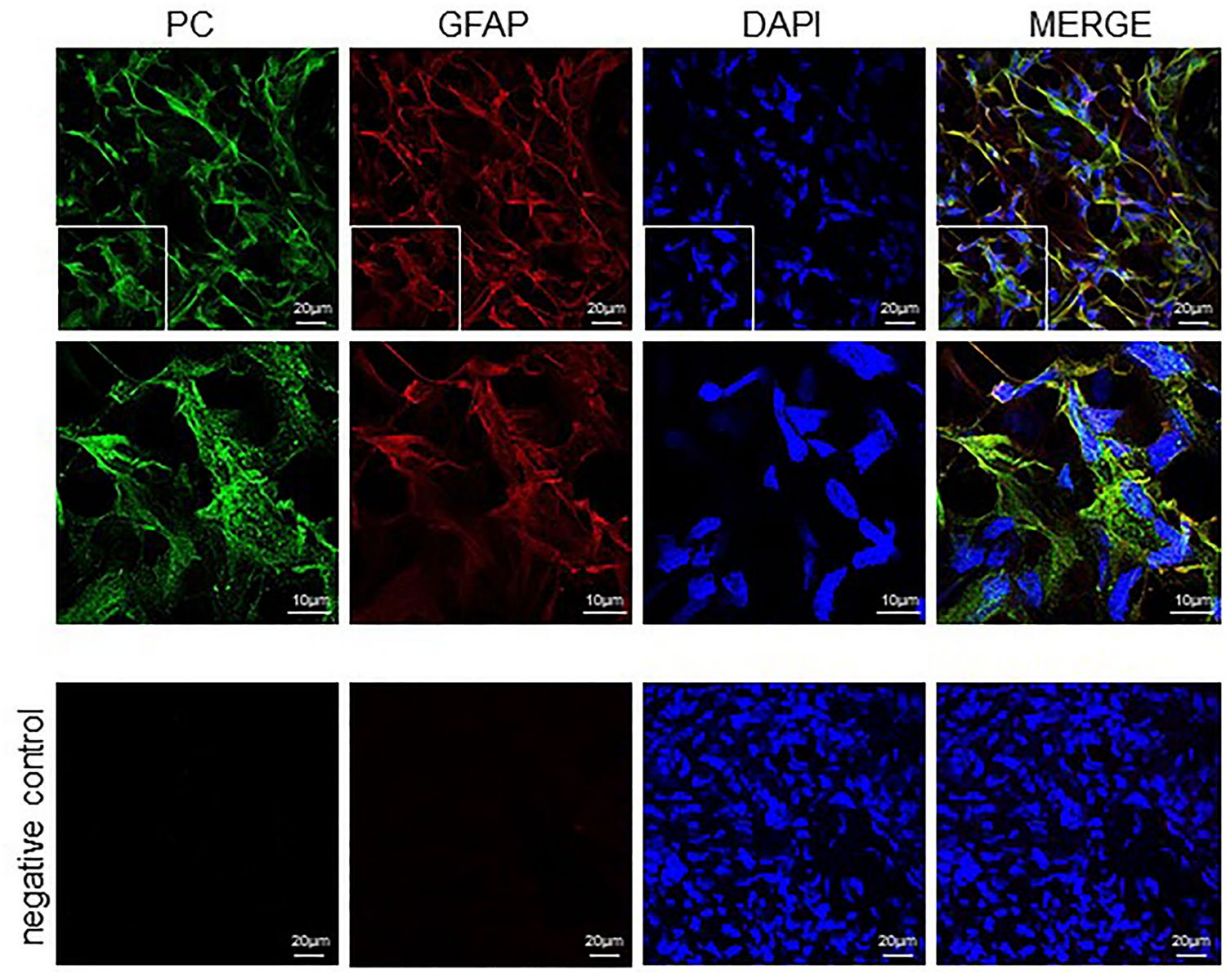
The presence of pyruvate carboxylase in glioblastoma. The pyruvate carboxylase (PC) was immunohistochemically labeled with set of antibodies and appear as a green and counterstained with anti-GFAP (red) and DAPI (blue). To better visualize the distribution of PC in the glioblastoma section, the photomicrographs with magnified parts are shown. The corresponding scale bars are indicated

**Fig. 5:**
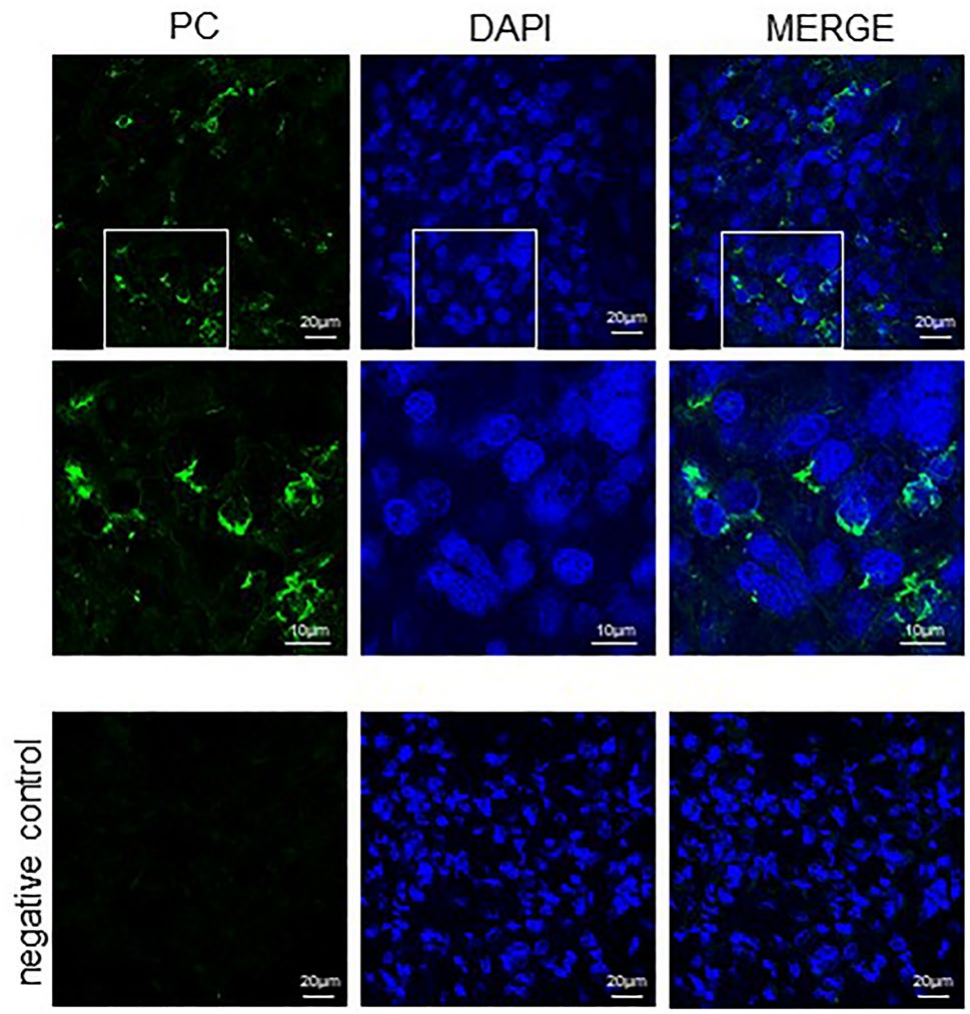
The presence of pyruvate carboxylase in astrocytoma. The pyruvate carboxylase (PC) was immunohistochemically labeled with set of antibodies and appear as a green and counterstained with DAPI (blue, nuclei). To better visualize the distribution of PC in astrocytoma section, the pictures of higher magnification are shown. The corresponding scale bars are indicated

Immunofluorescence method was applied to visualize the expression of PC among the cells in glioblastoma (Fig. 4, PC) and in astrocytoma (Fig. 5, PC) sections. Three samples of different glioblastoma and astrocytoma samples were analyzed for immunohistochemistry and one sample of them is presented. In sections of both tumors types the green fluorescence signal was visible when observed by fluorescence microscopy. Indeed, mouse anti-glial fibrillary acidic protein (GFAP) IgG molecules in combination with the secondary antibodies, which were conjugated to Alexa fluor 633 (red fluorescence signal), provided identification of GFAP positive cells (Fig. 4, GFAP). The colocalization of GFAP and PC positive signals appeared in yellow (Fig. 4, Merge). DAPI intercalating fluorochrome was used to visualized the cell nuclei (Figs. 4, DAPI; 5, DAPI). In the case of the negative controls, when only the mixture of secondary antibodies was applied, the fluorescent signal was not observable (Figs. 4, 5 negative control). For this reason, it can be expected that the observed fluorescence signal corresponds with the presence of PC in the sections from glioblastoma and astrocytoma.

## Discussion

With the aim to assign the expression of PC among different types of human brain tumors, we tested the presence of PC in protein extracts derived from the surgically obtained specimens from human glioblastoma, astrocytoma, oligodendroglioma, and meningioma tumors. The applied immunodetection methods on tumor homogenate proteins revealed the expression of PC in all tested samples. These results provide the hint that PC could be ubiquitously expressed in human glioblastoma, astrocytoma, oligodendroglioma, and meningioma tumors (Figs. 2, 3). Furthermore, the Western blot analysis shows that PC is also present in the human brain and meninges forming cells (Fig. 2).

We assume that the immunoblotting signal for PC is higher in the tumors compared to the healthy samples due to the higher demands of tumor cells for anaplerotic metabolism to support their rapid growth and multiplication [27]. The increased anaplerotic capacity of tumor cells facilitated by PC, might support the increased demand for synthesis of fatty acid and cholesterol [19–21]. These biosynthetic processes require the supply of acetyl-CoA in cytosol. In this respect, the withdrawal of mitochondria-born citrate could be employed. Therefore, contributing of PC to increase the anaplerotic capability of tumor cells could prevent the collapse of the Krebs cycle and/or mitochondiral metabolism [21].

The presented results of immunofluorescent experiments show that the PC is expressed to different extents among glioblastoma and astrocytoma tumor-forming cells (Figs. 4, 5). The fluorescent signal of PC is localized in the proximity of the cell nuclei and observable in the vast majority of glioblastoma-forming cells and a significant proportion of astrocytoma cells. The levels of PC can vary in different ranges from tumor to tumor and at the same time in the same types of tumor from patient to patient as we showed in dot blot (Fig. 3) and also in immunocytochemistry (Figs. 4, 5). It was shown that GFAP expression in glioblastoma tumors is increased in comparison to other brain cancer types [28]. Indeed, GFAP-positive cells accounted for the majority of the glioblastoma-forming cells (Fig. 4).

The expression of PC was already confirmed in several other human cancer types, e.g., small cell lung cancer [13], breast [14], colorectal [29], pancreatic [30], and prostate [31] cancers. The complementary studies with either cultured cancer cells or on animal models revealed that the anaplerotic role of the PC supports several cellular processes [15, 25, 32] with an impact on the growth [17], progression [29], resistance, and metastatic capability [14, 25] of tumors.

**Fig. 6:**
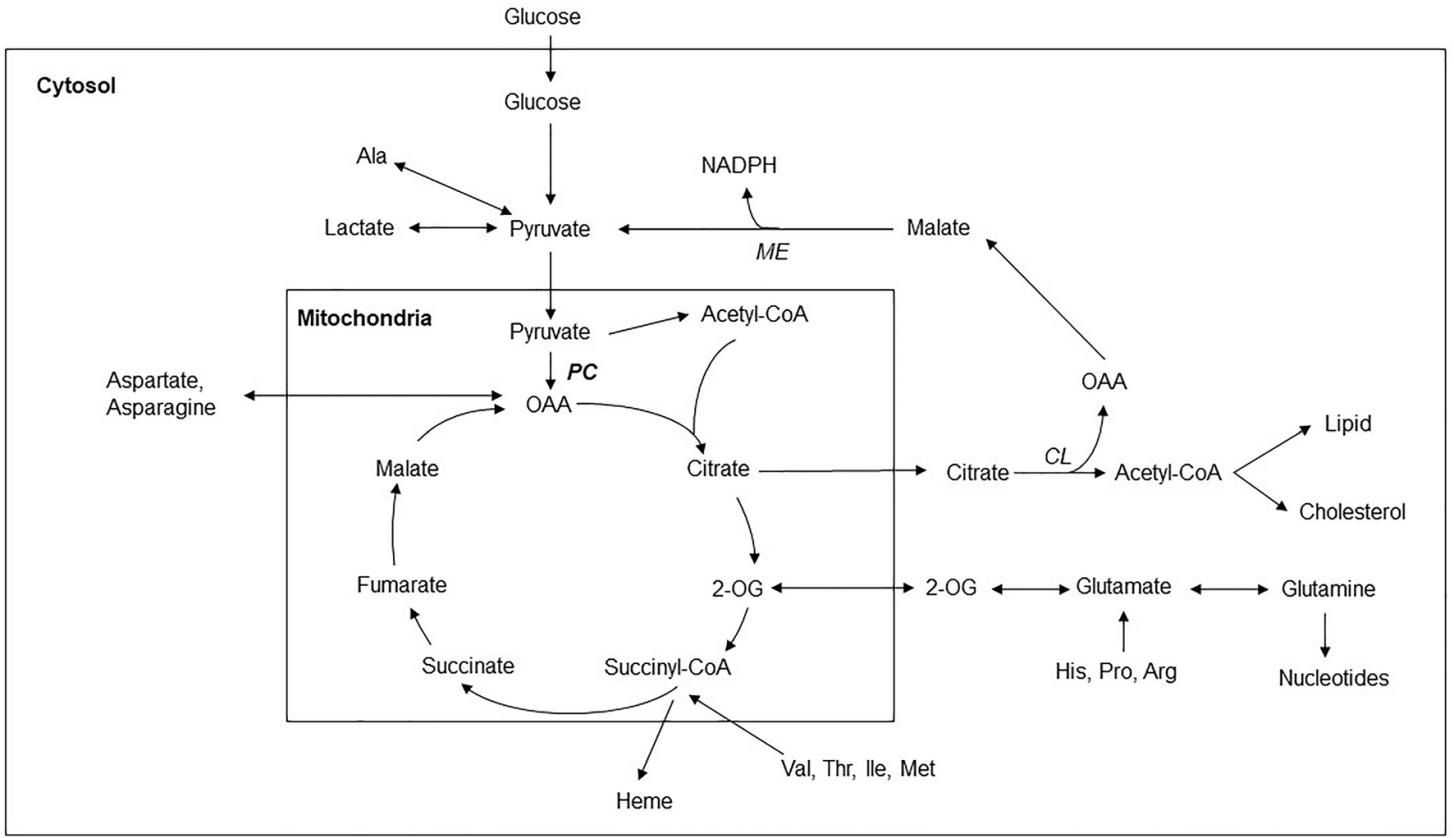
Anaplerotic role of pyruvate carboxylase (*PC*) in brain cancer cells. Glucose and amino acids may serve the roles of the primary source of energy and carbon atoms for biosynthetic reactions in brain cancer cells. Glucose enters the glycolytic pathway and is metabolized to pyruvate. In cancer cells, the pyruvate is mainly reduced to lactate by lactate dehydrogenase and, in such a way, could contribute to regenerating the cytosolic pool of NAD^+^. In addition, pyruvate molecules could be either reversibly transaminated to alanine or imported into a mitochondrial compartment, in which pyruvate might be converted to acetyl-CoA or oxaloacetate (OAA). Conversion of pyruvate to oxaloacetate by pyruvate carboxylase (PC) is one of the possible anaplerotic reactions. The others are: (i) converting glutamine and glutamate to 2-oxoglutarate (2-OG); (ii) generating OAA from asparagine and aspartate; (iii) synthesis of succinyl-CoA by catabolism of several compounds including Val, Thr, Ile, and Met. Withdrawal of the TCA intermediates from mitochondria into the cytosol might be essential for sustaining the anabolic metabolism in cancer cells, e.g., the synthesis of fatty acids and cholesterol

The anabolic metabolism of cancer cells is dependent on the cellular capability to restore the levels of the intermediates of the Krebs cycle (Fig. 6) by anaplerotic processes[33]. Only a limited number of reactions is considered to possess the anaplerotic role. The most important anaplerotic process in cancer cells is the generation of 2-oxoglutarate from extracellularly originating glutamine. In this respect, the catabolism of glutamine, which is taken up from the cellular milieu, could impart the cells with both carbon skeleton and reduced nitrogen. Increased consumption of glutamine by different brain tumors, including glioblastoma, was documented by a previous metabolomic study [34]. The recent studies with the cultured cancer cells and animal models revealed that the anaplerotic role of PC could significantly participate in sustaining the metabolism and survival of cancer cells and the growth of tumors, especially if glutamine supply is insufficient [11]. The remaining three anaplerotic processes include the conversion of propionyl-CoA to succinyl-CoA and aspartate to oxaloacetate or fumarate through either adenylosuccinate or fumarylacetoacetate. However, the importance of those remaining processes for the metabolism of cancer cells has not been fully elicited yet.

PC expression in human tumors is a prerequisite for the cells to explore its enzymatic possibility. The regulation of the PC expression is rather complex [35], and includes converting apoenzyme to its active biotinylated holoenzyme form. The disappearance of the immunoprobing signal of PC from the glioblastoma homogenate treated with streptavidin indicates that the PC is present in biotinylated (Fig. 1), enzymatically active form. Deprivation of glutamine could also promote increased PC activity and, therefore, could support the independence of cellular anaplerotic metabolism on extracellularly available glutamine [11]. Since several approaches to target the glutamine metabolism of cancer cells are under investigation and testing [7, 8], the possibility of cancer cells to supplement or substitute the anaplerotic role of glutamine by PC facilitated anaplerotic metabolism might be taken into account. In this respect, the knowledge about the expression of PC in particular types of tumors could contribute to proposing and designing the therapeutic approaches affecting cancer cells characteristics by altering their metabolism [36].

## Methods

The Ethics Committee of the Jessenius Faculty of Medicine CU in Martin has approved the experiments with human samples under the code FK 75/2018. Informed consent was obtained from all subjects of this study and all participants have been performed in accordance with the Declaration of Helsinki. The selected characteristics of the patients, which are donors' brain tumor specimens and who had not previously received radiotherapy or chemotherapy, are in Table 1.

All biopsy tissues obtained during tumor resection were provided by Department of Neurosurgery (Roosevelt Hospital, Banská Bystrica, Slovakia) between November 2018 and September 2019 and were histologically confirmed according to the criteria of the used WHO Classification [37].

### Homogenization and Production of Protein Extracts

For experiments were used ten samples of glioblastoma, ten of astrocytoma, seven of oligodendroglioma, and seven of meningioma obtained by biopsies. Immediately after surgery, the tumor was subdivided into several pieces and stored at − 80 °C. The tumor samples were used to prepare homogenates in the buffer consisting of 0.3 M sucrose, 5 mM EDTA, 0.3 mM phenylmethylsulfonyl fluoride, and 30 mM K_2_HPO_4_,/KH_2_PO_4_ with pH 7.0; according to a method already described [38].

### Extraction of Biotin-Containing Proteins

The clarified glioblastoma homogenate was used to generate two fractions, either free or enriched with the biotin-containing proteins. The level of glioblastoma homogenate proteins was set to 1 µg/ml with Dulbecco’s Phosphate Buffered Saline (DPBS; D8537, Sigma). The biotin-containing proteins were extracted from lysates by the already used method [38]. Briefly, 150 µl of diluted lysate was incubated with 500 µl of streptavidin-agarose beads suspension (Thermo-Fisher) at room temperature for one hour. Subsequently, the suspension was centrifuged at 3000×*g* for 5 min, and supernatant-1 was collected. The sediment was washed twice with DPBS to remove unspecifically interacting proteins; those were collected and combined with supernatant-1. The generated supernatant-1 was depleted of biotin-containing proteins. The biotin-containing proteins were eluted from a complex with streptavidin agarose beads with DPBS supplemented with biotin (100 µg/ml). During incubation at room temperature for 30 min. After centrifugation of suspension at 3000×*g* for 5 min, supernatant-2 was collected. The proteins in the diluted glioblastoma homogenate, supernatant-1, and supernatant-2, were precipitated with acetone and collected by centrifugation at 12,000×*g* for 12 min. Supernatants were discarded, and air-dried pellets were resuspended in DPBS and used for further analysis.

### Dot Blot Analysis

The clarified homogenates derived from brain and glioblastoma, astrocytoma, oligodendroglioma, and meningioma tumors were used to evaluate the expression of PC by adaptation of previously described dot blot method [38]. Briefly, 0.5 µg of protein were spotted on nitrocellulose membrane, and was probed with rabbit antiserum against pyruvate carboxylase diluted 1:200 in blocking solution overnight. The blocking solution (BS) consisting of Tris-buffered saline (TBS; 19.8 mM Tris, 136 mM NaCl, with pH adjusted to 7.5) supplemented 2% (W/W) albumin (BSA; Applichem) and 0.05% (W/V) Tween-20 (Sigma). Applied secondary antibodies were goat anti-rabbit IgG conjugated with horse-radish peroxidase diluted in BS 1:10,000. The chemiluminescent signal was recorded and subsequently the intensity of chemiluminescent signals was quantified by the Image Studio Lite version 5.2 software (LI-COR Biotechnology). The final values represent the intensity of the specific signal that was calculated by subtracting the background value from signal of the sample.

### Cell Culturing and Preparation of Cell Lysate

For the study, we used human HepG2 hepatocarcinoma cell line (ATCC-HB-8065; DSMZ). The cells were cultured according to the supplier’s instructions.

The cells grown to 80% confluency were washed twice with ice-cold DPBS and subsequently, cells were lysed in ice-cold lysis buffer consisting of 30 mM TRIS with pH 7.6, supplemented with 150 mM NaCl, 1% (W/V) CHAPS (Sigma), and with 1% (V/V) of commercially available Halt™ protease inhibitor (Thermo-Scientific). Centrifugation at 10,000×*g* for 10 min was used for clarification of lysates. The generated supernatants were aliquoted and stored at − 20 °C before used.

### Western Blot Analysis

The proteins were resuspended in loading buffer (300 mM TRIS, 300 mM dithiothreitol, 60% V/W glycerol, 0,3% V/W bromphenol blue, pH 6.8) were denatured by boiling for 5 min before they were loaded on 8% acrylamide gels for separation by SDS-PAGE method [39]. Separated proteins were electroblotted on the nitrocellulose membrane (Bio-Rad Laboratories), which were subsequently incubated in the BS at room temperature for one hour. The primary antibodies, rabbit anti-PC, were diluted in BS to a final ratio of 1:500 at 4 °C for overnight. After three washing steps, the solution of mouse anti-rabbit conjugated with POD in BS (final dilution ratio 1:10,000) was applied. After one hour of incubation at room temperature, the membrane was washed three times, and subsequently, a chemiluminescent signal was developed after soaking in SuperSignal West Pico Chemiluminescent Substrate solution (Thermo-Scientific) and recorded by Chemidoc XRS system (Bio-Rad Laboratories). Two loading controls were performed by visualization of β-actin (Santa Cruz Biotechnology) and GAPDH (Santa Cruz Biotechnology).

### Immunohistochemistry

Out of the collected tumor samples were randomly selected glioblastoma and astrocytoma those were used to prepare 15 μm thick cryosections on Shannon Cryotome E (Thermo-Scientific Waltham). The sections collected on SuperFrost Plus (VWR International) glass microscope slides were left to air dry. After that, the sections were fixed by immersion in methanol for 10 min. The blocking-solution 2 (BS2), consisting of PBS supplemented with 1% (V/W) Triton X-100 (Sigma) and 2 mg/ml BSA, was layered on sections to prevent the unspecific protein binding activity for 60 min. Subsequently, the sections were incubated at 4 °C for overnight with a mixture of primary antibodies that were diluted in BS2: rabbit anti-PC, 1:250 and mouse anti-GFAP, 1:250. Prior to the application of secondary antibodies, the sections were washed 3 × 10 min in TBS enriched with 1% (V/V) Triton X-100. Secondary antibodies: goat anti-rabbit linked with Alexa Fluor 488 (1:1000; Invitrogen) and goat anti-mouse linked with Alexa Fluor 633 (1:1000, Invitrogen) were mixed in BS2 and were applied for 90 min. Subsequently, slides were washed three times in PBS. During the second washing step, PBS was supplemented with 5 µg/ml DAPI (Sigma) to visualize the cell nuclei. Finally, slides were rinsed with H_2_O and mounted with Fluoroshield Mounting Medium (Sigma). Only secondary antibodies were used for preparation of negative controls.

### Microscopic Analysis

For imaging samples from glioblastoma and astrocytoma frozen sections a Zeiss Axio Examiner/LSM 880 confocal system (Carl Zeiss, Jena, Germany) with a Zeiss Plan-Apochromat 40 × /1.3 Oil DIC M27 objective and Zeiss Zen Black software was used. The 405 nm diode was used to visualize the cell nuclei (DAPI; A_max_ = 359 nm and E_max_ = 457 nm); argon 488 nm laser (Alexa Fluor 488; A_max_ 494 nm, E_max_ 519 nm) was used to visualize the PC and the 633 nm laser to visualize GFAP in samples (Alexa Fluor 633; A_max_ = 621 nm, E_max_ = 639 nm). Images were acquired with a resolution of 2048 × 2048 pixels, 1388 × 1040 pixel size and with a pinhole of 1.0 AU. A 2.5 × zoom was used for a more detailed view. The resulting images were processed using Zeiss Zen Blue software (Zeiss).

### Statistical Analysis

The results are presented as mean ± SEM. The analysis of variance (ANOVA) test was performed to test the difference among the obtained values. The statistical analysis was performed with the software GraphPad Prism version 9.0.2 (GraphPad Software).

## Supplementary Information

Below is the link to the electronic supplementary material.

Supplementary file1 (PPTX 5486 kb)

## Data Availability

All data generated or analysed during this study are included in this published article [and its supplementary information files]. Enquiries about data availability should be directed to the authors.
