# Supplementary material for: Immunodetection of Pyruvate Carboxylase Expression in Human Astrocytomas, Glioblastomas, Oligodendrogliomas, and Meningiomas
Source: Neurochem Res. 2023 Jan 20;48(6):1728–36. doi: 10.1007/s11064-023-03856-5 (PMC10119210; doi:10.1007/s11064-023-03856-5)
Supplement: Supplementary file 1 — Supplementary file1 (PPTX 5486 kb) [file 11064_2023_3856_MOESM1_ESM.pptx]

## Slide 1
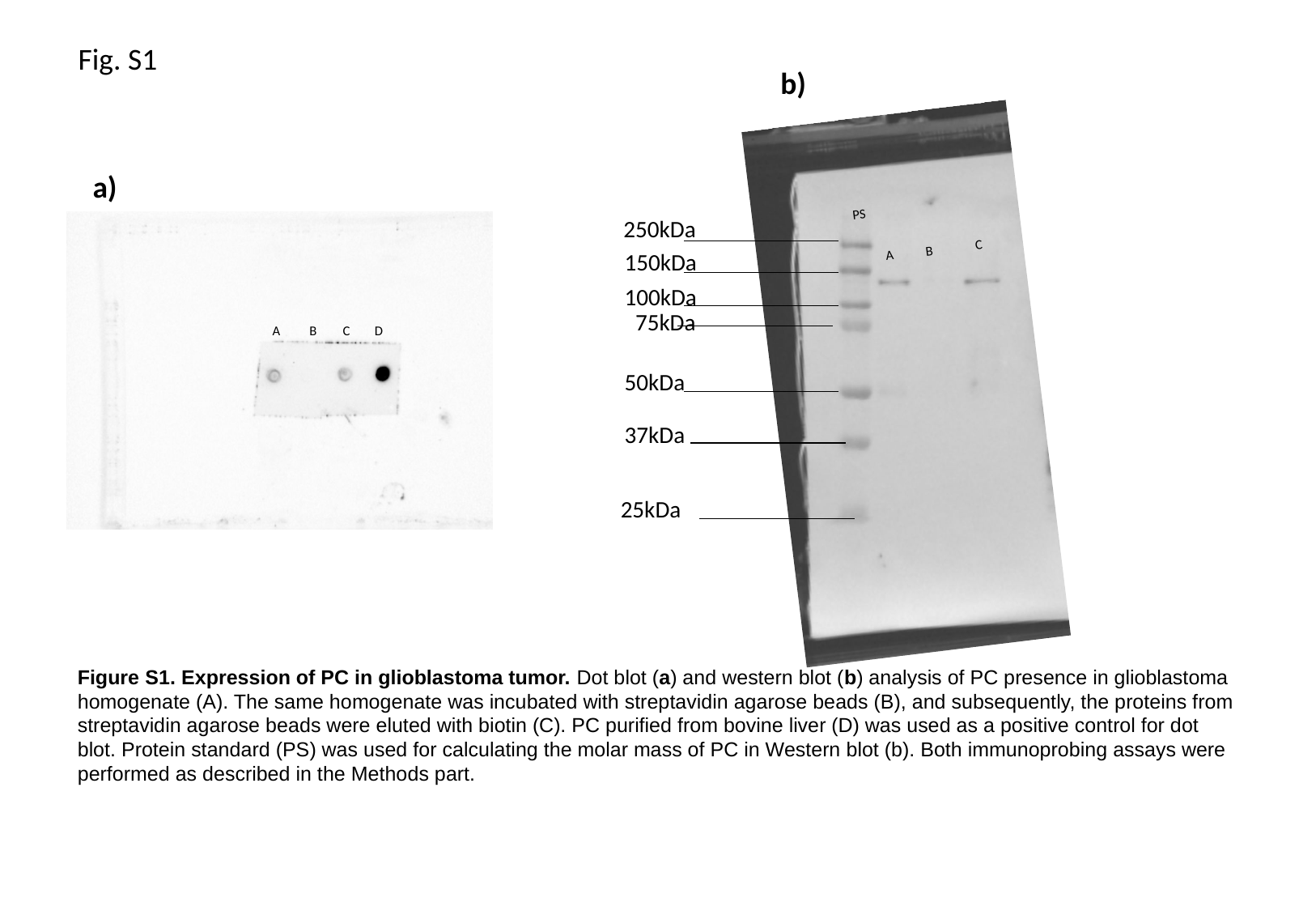

Fig. S1
b)
PS
A
B
C
a)
250kDa
150kDa
100kDa
75kDa
A
B
C
D
50kDa
37kDa
25kDa
Figure S1. Expression of PC in glioblastoma tumor. Dot blot (a) and western blot (b) analysis of PC presence in glioblastoma homogenate (A). The same homogenate was incubated with streptavidin agarose beads (B), and subsequently, the proteins from streptavidin agarose beads were eluted with biotin (C). PC purified from bovine liver (D) was used as a positive control for dot blot. Protein standard (PS) was used for calculating the molar mass of PC in Western blot (b). Both immunoprobing assays were performed as described in the Methods part.

## Slide 2
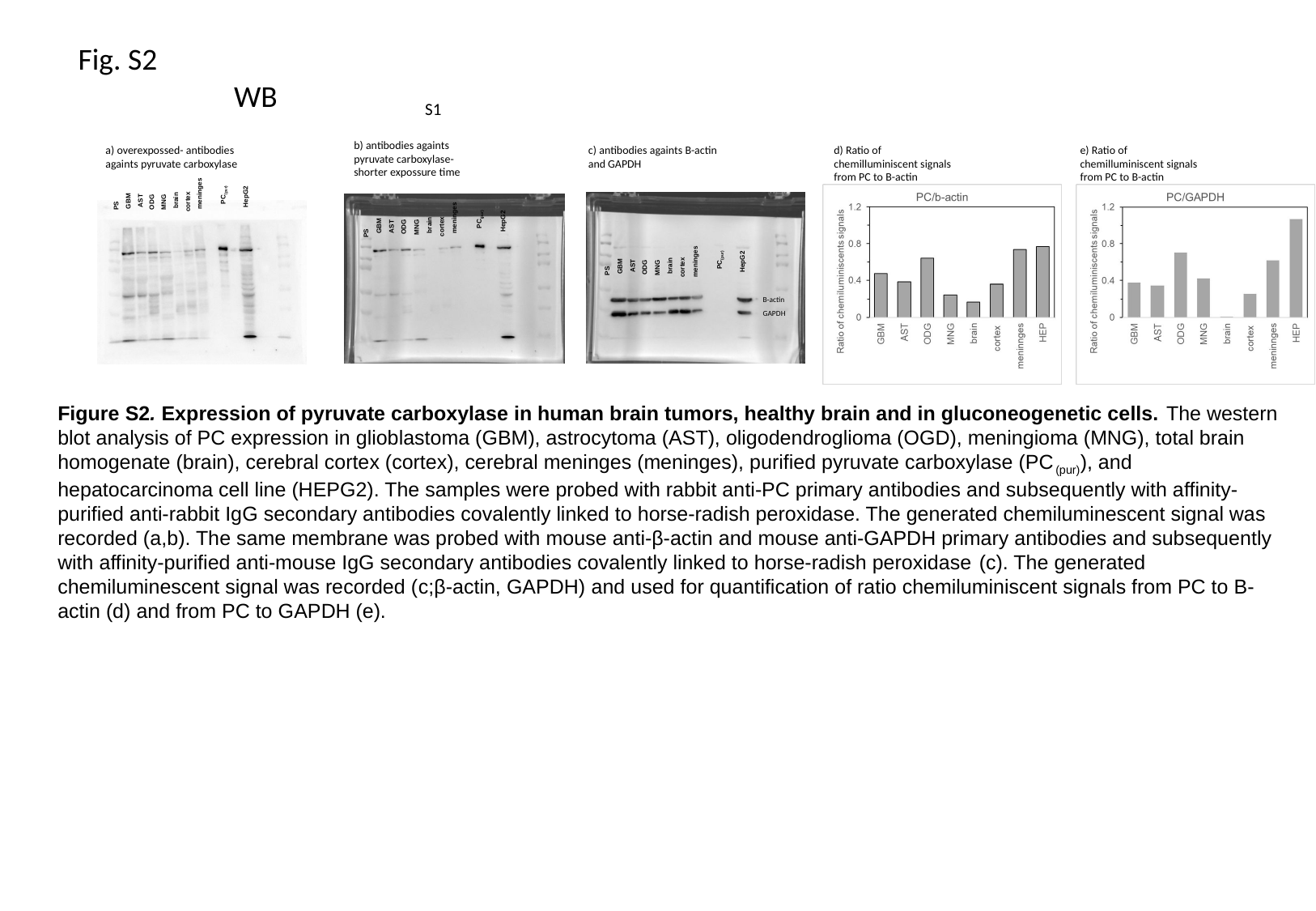

Fig. S2
WB
S1
b) antibodies againts pyruvate carboxylase- shorter expossure time
a) overexpossed- antibodies againts pyruvate carboxylase
c) antibodies againts B-actin and GAPDH
d) Ratio of chemilluminiscent signals from PC to B-actin
e) Ratio of chemilluminiscent signals from PC to B-actin
meninges
PC(pur)
HepG2
cortex
brain
GBM
AST
ODG
MNG
PS
meninges
PC(pur)
HepG2
cortex
brain
GBM
AST
ODG
MNG
PS
B-actin
GAPDH
meninges
PC(pur)
HepG2
cortex
brain
GBM
AST
ODG
MNG
PS
Figure S2. Expression of pyruvate carboxylase in human brain tumors, healthy brain and in gluconeogenetic cells. The western blot analysis of PC expression in glioblastoma (GBM), astrocytoma (AST), oligodendroglioma (OGD), meningioma (MNG), total brain homogenate (brain), cerebral cortex (cortex), cerebral meninges (meninges), purified pyruvate carboxylase (PC(pur)), and hepatocarcinoma cell line (HEPG2). The samples were probed with rabbit anti-PC primary antibodies and subsequently with affinity-purified anti-rabbit IgG secondary antibodies covalently linked to horse-radish peroxidase. The generated chemiluminescent signal was recorded (a,b). The same membrane was probed with mouse anti-β-actin and mouse anti-GAPDH primary antibodies and subsequently with affinity-purified anti-mouse IgG secondary antibodies covalently linked to horse-radish peroxidase (c). The generated chemiluminescent signal was recorded (c;β-actin, GAPDH) and used for quantification of ratio chemiluminiscent signals from PC to B-actin (d) and from PC to GAPDH (e).

## Slide 3
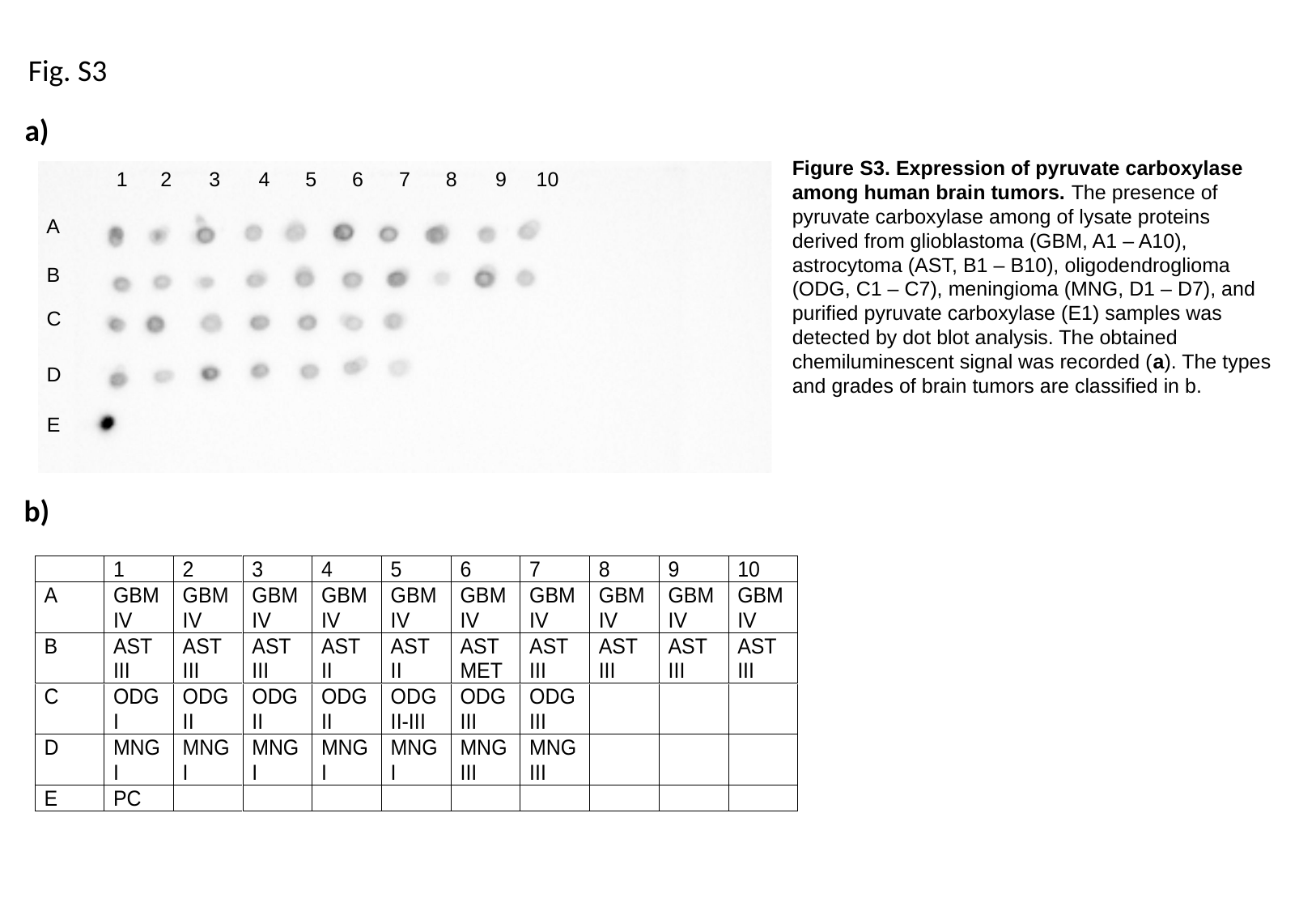

Fig. S3
a)
Figure S3. Expression of pyruvate carboxylase among human brain tumors. The presence of pyruvate carboxylase among of lysate proteins derived from glioblastoma (GBM, A1 – A10), astrocytoma (AST, B1 – B10), oligodendroglioma (ODG, C1 – C7), meningioma (MNG, D1 – D7), and purified pyruvate carboxylase (E1) samples was detected by dot blot analysis. The obtained chemiluminescent signal was recorded (a). The types and grades of brain tumors are classified in b.
1
2
3
4
5
6
7
8
9
10
A
B
C
D
E
b)
